# How important are quantum mechanical effects in controlling biological functions: Enzymes, electron transfer and bird navigation

**DOI:** 10.1002/pro.70683

**Published:** 2026-06-19

**Authors:** Aoxuan Zhang, Arieh Warshel

**Affiliations:** ^1^ Department of Chemistry University of Southern California Los Angeles California USA

**Keywords:** quantum biology, enzyme catalysis, nuclear quantum effect, electron transfer, QM/MM, bird navigation

## Abstract

In light of the centennial of the Schrödinger equation, this article addresses the popular idea that quantum mechanical effects play an important role in biological systems. We start by defining what is qualified as a quantum effect. We then clarify the idea that quantum mechanical tunneling is a crucial factor in enzyme catalysis. Here we show that quantum tunneling is in fact anticatalytic and that there is no consistent theoretical and experimental evidence that the tunneling effects are enhanced significantly by enzymes relative to the corresponding reference solution reactions. We next turn to electron transfer reactions, arguing that in most cases the efficiency of the reaction is controlled by classical effects. We also consider the low barrier hydrogen bond idea and clarify its anticatalytic nature. In addition, we present a specific case study of electron transfer in a protein system potentially responsible for bird navigation, providing a concrete example for evaluating the role of quantum and classical effects under biologically relevant conditions. We argue that for the electron transfer step in this system, the most important control is associated with the classical control of the relevant potential surfaces and the fluctuations of the key energy gaps.

## 1 WHAT CAN WE LEARN FROM SPECIFIC CASES

Quantum mechanics is the basis of the determination of molecular potential surfaces and the corresponding forces. This fact should be distinguished from the existence of quantum mechanical effects which are effects that cannot be described by classical mechanics. As much as biological molecules are concerned the issue is how important are quantum mechanical effects in biological systems. In addressing this question, we have to recognize the natural inclination to assume that complex biological processes are guided by quantum mechanical factors (e.g., tunneling). This issue is analyzed carefully below.

### 1.1 The role of QM calculations

Before we start considering the possible role of quantum effects in biology, it is useful to consider the undeniable role of quantum mechanical calculations in studies of biological functions. Most notable are QM/MM calculations of enzymatic reactions (Gelfand & Warshel, 2025; Kamerlin et al., 2009; Nandi & Warshel, 2025) and other processes (Nandi et al., 2024; Senn & Thiel, 2009) that have been proven to be crucial for understanding the molecular origin of biological functions. For example, it is basically impossible to determine the origin of the catalytic effect of enzymes without some type of QM/MM calculation (Kamerlin & Warshel, 2011). However, this does not mean that we are dealing with quantum mechanical catalytic effects. That is, quantum mechanical calculations are crucial for obtaining potential energy surfaces for molecular processes. However, this does not mean that we are dealing with a quantum mechanical effect. In our view, quantum mechanical effects are effects that cannot be described by classical mechanics treatments of the motion on the corresponding Born Oppenheimer surfaces. In fact, for the purpose of this paper it is not useful to discuss the importance of quantum mechanical effects in biology without defining what we mean by such effects.

It seems to us that effects that can be described by propagating molecular dynamics (MD) trajectories on Born‐Oppenheimer surfaces should not be considered as quantum mechanical effects. In contrast, nuclear quantum effects (NQE) refer specifically to phenomena associated with the quantum nature of nuclear motion that cannot be described by classical mechanics. These include effects such as zero‐point energy and quantum tunneling. In the following sections, we examine whether such effects play a significant functional role in biological systems.

### 1.2 Enzyme catalysis

It has been very appealing to suggest that enzyme catalysis is associated with quantum effects (Marais et al., 2018). Such problematic implications are usually done by invoking terms like “coupling,” “tunneling,” “Coherence,” and more. However, without any successful demonstration that the alleged quantum effects actually lead to a specific observed catalytic effect, it has to take such allegations seriously. In the sections below, we will examine some of the most prevalent proposals.

#### 1.2.1 There is no consistent evidence that quantum tunneling effects contribute to enzyme catalysis

**FIGURE 1:**
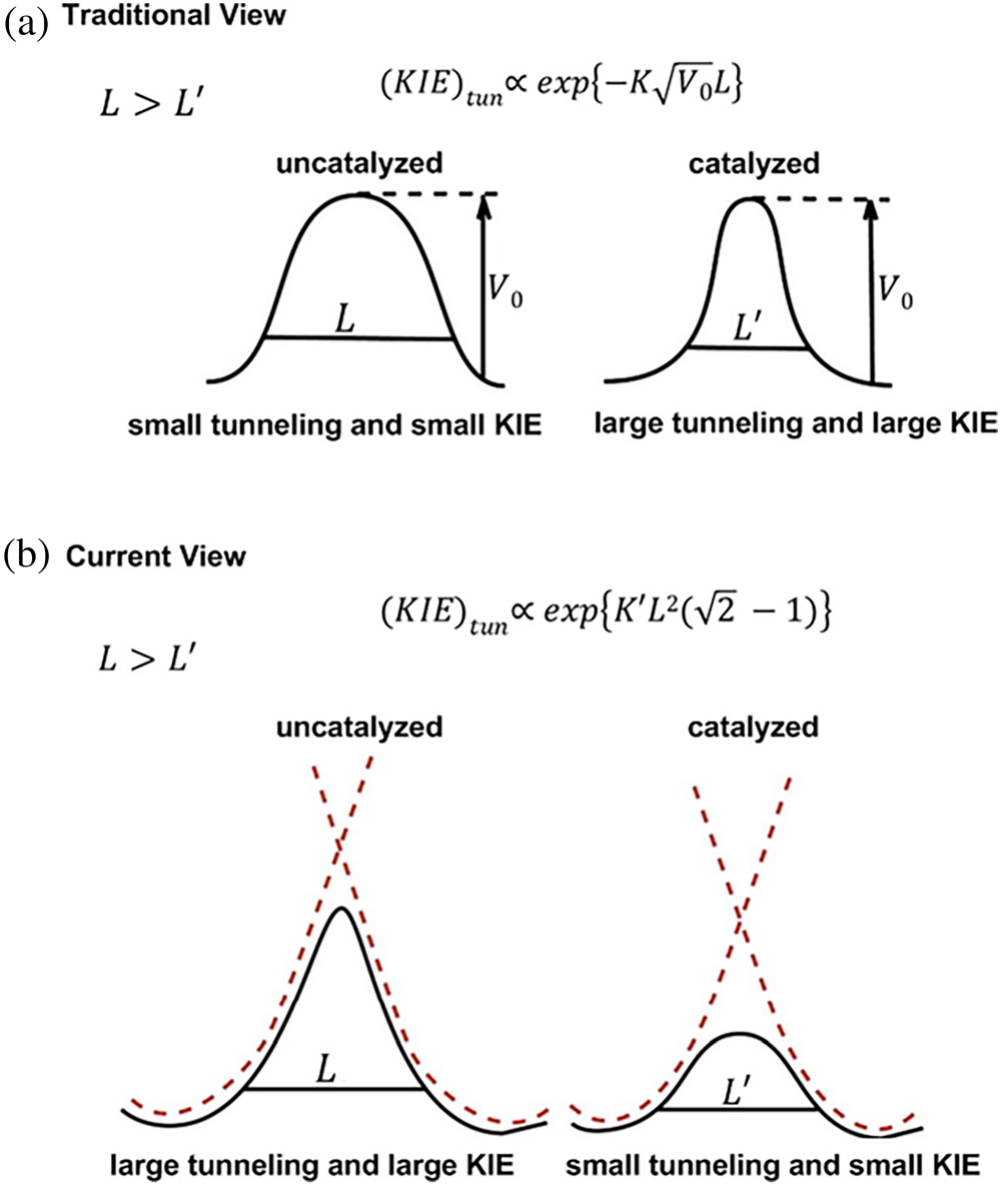
Schematic illustration of the anticatalytic dependence of nuclear quantum mechanical (NQM) effects on the donor–acceptor distance in hydride transfer reactions. Solid black curves represent potential energy along the reaction coordinate, and L represents the thickness of the corresponding nuclear tunneling path. Panel (a) presents the conventional (incorrect) picture, in which compression of the donor–acceptor distance is assumed to enhance NQM contributions by keeping barrier height unchanged but narrowing the barrier thickness, and thereby increase tunneling in enzyme catalysis. Panel (b) summarizes our more realistic (correct) interpretation, based on extensive simulations and theoretical analysis, showing that NQM contributions instead decrease as the donor–acceptor distance is reduced, since compression leads to barrier disappear and renders the reaction effectively classical. Adapted from (Liu & Warshel, 2007).

One of the most popular arguments that quantum mechanical effects play an important role in biology involves the idea that quantum mechanical tunneling leads to large catalytic effects. Apparently, this is a very appealing idea, as it implies that the enzyme compresses the donor acceptor distance, creating a narrower potential barrier and large tunneling (Bahnson et al., 1997; Ball, 2004; Klinman, 1989; Luo et al., 1999; Mincer & Schwartz, 2003; Sutcliffe & Scrutton, 2000). However, our studies (Liu & Warshel, 2007) established that the nuclear quantum mechanical (NQM) effects decrease rather than increase due to compression. That is, when the distance between the donor and acceptor is sufficiently compressed, the mixing between the two electronic states makes the adiabatic surface very flat rather than narrow, so that the tunneling effect decreases (see Kamerlin et al., 2010 and Figure 1).

The analysis of Figure 1 has important implications for any proposals that attribute a significant role to NQM in enzyme catalysis. Specifically, the factors that increased NQM contributions are found to be anticatalytic. This behavior arises primarily because the rate constant decreases as the donor–acceptor distance increases, whereas the tunneling increases when the donor acceptor distance increases. The observation that a large kinetic isotope effect (KIE) reflects an increase, rather than a decrease, in the donor–acceptor distance is counterintuitive and has therefore been a persistent source of confusion (see, e.g., Kamerlin &`Warshel, 2010b for a detailed discussion). In our view, a central difficulty with the catalytic tunneling proposal is the fact that, despite our demonstration of the fundamentally anticatalytic nature of the original tunneling picture, namely, donor–acceptor compression reduces rather than enhances tunneling. At this point, it may be useful to further expand on the argument that the tunneling proposal is anticatalytic by pointing out that the tunneling would increase when the donor acceptor distance is forced to increases. At this situation, the rate constant decreases since the barrier becomes higher despite the tunneling correction. The above issues have not been addressed by proponents of catalytic tunneling and a reasonable response is unlikely to emerge.

Kohen and coworkers (Kohen et al., 1999) observed that the activation enthalpy (ΔH^‡^) for the reaction catalyzed by thermophilic alcohol dehydrogenase (ADH) decreases from 23.6 kcal mol^−1^ at lower temperatures (0–30°C) to 14.6 kcal mol^−1^ at higher temperatures (30–65°C). They interpreted this behavior as evidence for a contribution to k_cat_ arising from vibrationally enhanced tunneling at elevated temperatures. Although this interpretation emphasized dynamical effects, we have demonstrated (Liu & Warshel, 2007) that a substantial portion of the observed behavior—and clearly its dominant classical contribution—can be understood as an entropic effect, which can be rationalized by considering changes in the interactions between the reacting solute and its environment associated with variations in the polarity of the reacting atoms. Our analysis further indicates that the observed temperature dependence primarily reflects changes in the donor–acceptor distance. Consequently, the temperature trend implies that tunneling contributions are anticatalytic rather than catalytic (Liu & Warshel, 2007).

Overall, the arguments presented in Figure 1 have not been disputed by proponents of the catalytic tunneling proposal. Moreover, we are not aware of any case in which the tunneling effect simulated or measured in an enzyme significantly exceeds the corresponding effect in the reference solution reaction. Thus there is no real support for the use of tunneling in enzyme catalysis. Of course, to remove any confusion, we are not saying that enzyme reactions do not involve tunneling. What we are saying is that tunneling does not help with enzyme catalysis.

#### 1.2.2 Mode coupling

It has been very popular to suggest that enzymes work by mode coupling (Hammes‐Schiffer, 2004; Rod et al., 2003; Watney et al., 2003). Basically, this idea cannot have merit for enzyme catalysis, since the reference reaction in water involves solvent motions that can also be described as reactive modes, and thus the same effect is already present in the reference system, and hence those modes alone cannot contribute to enzymatic catalysis.

More specifically, a general analysis of the coupling between the protein (or solvent) vibrations and the chemical process was introduced by Warshel and coworkers in terms of the dispersed polaron (DP) spin boson treatment (Olsson & Warshel, 2004; Warshel & Parson, 2001).

The DP approach connects fluctuations of the EVB diabatic energy gap along MD trajectories to those of an equivalent harmonic system. The corresponding power spectrum for fluctuations in a given diabatic state is obtained from the Fourier transform of the energy‐gap autocorrelation function, yielding peaks at frequencies associated with modes coupled to the reaction. In the high‐temperature limit, the amplitudes of these peaks are proportional to the square of the displacement along the corresponding coordinates. Within this framework, it has been possible to reexamine several enzyme systems (Kurian et al., 2016), including some that have been cited as evidence for the importance of rate‐promoting modes in enzyme catalysis (Olsson & Warshel, 2004; Warshel & Parson, 2001).

The proponents of the mode coupling effects (e.g., Watney et al., 2003) claimed to identify networks of correlated conformational changes with components projected along the reaction path in simulations, but concluded that these correlations actually reflect equilibrium structural effects rather than genuine dynamical contributions. In general, the identification of correlated motions does not introduce any fundamentally new perspective on enzyme catalysis, since solvent reorganization along the reaction coordinate in solution likewise involves strongly correlated structural changes. Importantly, the EVB dispersed‐polaron (DP) analysis provides a consistent means to quantify the projection of protein motions onto the reaction coordinate and allows for a direct, quantitative comparison with the corresponding reference reaction in solution.

In enzymes, coupling between protein motions and the chemical process arises from fluctuating electrostatic interactions between the reacting solute and surrounding charged or polar residues, as well as bound water molecules. In solution, analogous effects originate from the reorientation of the solvation shells. In both cases, the reaction coordinate necessarily contains contributions from environmental (solvent) degrees of freedom. The essential distinction lies in the magnitude of the environmental coordinate changes during the reaction, which determines the reorganization energy and is generally smaller in enzymes due to active‐site preorganization. Consistent with this view, studies employing the DP framework (Kamerlin & Warshel, 2010a) have demonstrated that the mode‐coupling picture effectively amounts to expressing the reaction coordinate along a harmonic or quasiharmonic pathway. While this decomposition is useful for analyzing contributions to the reorganization energy, the catalytic effect itself originates from the reduced reorganization energy rather than from any time‐dependent coherence of specific modes.

#### 1.2.3 Entanglement and other problematic proposals

The idea that enzymes work by quantum entanglement can be popular for those who have not tried to truly understand how enzymes work. Such an idea has been proposed for example for the action of type II restriction endonucleases (Kurian et al., 2016). Presuming that the mechanism is unclear. However, the glaring problem with the proposal is that its proponents never tried to reproduce any observed parameters by their model or more importantly to see whether energy based models face any problem in reproducing the observed effects. For example, one should have examined if a reasonable free energy landscape, probably with a kinetic equation, can reproduce the observed time dependence. Using arbitrary claims is not likely to be scientifically useful. Otherwise, one can suggest that the action of restriction enzymes is modulated by gravitational waves. The actual proposal involves an arbitrary phenological Hamiltonian that is not based on any clear energy consideration.

An equally problematic proposal has involved the strange speculation that epigenetics involves quantum effects (Siebert et al., 2023). It is regrettable that such proposals are not met by more serious scrutiny with the requirement of reproducing some observed effects.

### 1.3 Low‐barrier hydrogen bond (LBHB) and covalent catalysis

**FIGURE 2:**
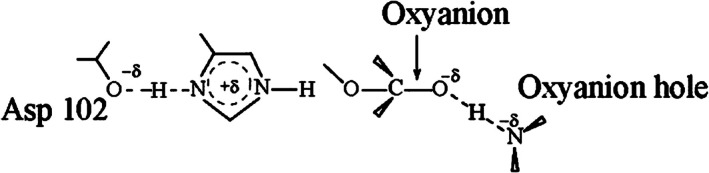
Schematic illustration of the low‐barrier hydrogen bond (LBHB) proposal in serine proteases. The figure depicts a covalent interaction model—whose validity remains to be established—in which active‐site groups, such as Asp102 and the oxyanion hole, form a partial covalent bond with the transition state of the reacting system, both in the enzyme and in solution. Incorporation of environmental effects into the energies of the diabatic states enables a quantitative assessment of how changes in the surroundings influence the charge‐transfer (CT) character of the hydrogen bond. This EVB‐based approach has been applied in quantitative analyses of putative hydrogen bonds to the environment (Garcia‐Viloca et al., 2001; Mulholland et al., 2000) and has shown that the LBHB hypothesis cannot account for the catalytic effect of preorganized hydrogen bonds.

A widely discussed proposal for the origin of enzyme catalysis is the low‐barrier hydrogen bond (LBHB) hypothesis (Cassidy et al., 1997; Cleland & Kreevoy, 1994; Frey et al., 1994; Pan & McAllister, 1997). This proposal is based on the assumption that the formation of a partially covalent interaction between enzyme hydrogen bonds and the transition state of the reacting system leads to catalytic enhancement (Cassidy et al., 1997; Pan & McAllister, 1997). Because it invokes direct bonding interactions with the enzyme, the LBHB hypothesis may be viewed as an appeal to a quantum mechanical effect. However, this proposal has not been evaluated using rigorous energetic considerations. The primary distinction between the LBHB picture and the well‐established view that preorganized hydrogen bonds stabilize the transition state electrostatically (Warshel & Aqvist, 1991) is that an LBHB is assumed to involve a partially covalent, delocalized bond, such as a bond of the form X–H–Y, where Y may represent, for example, a negatively charged oxygen atom of the solute in the transition state (see also Figure 2).

Warshel and Papazyan (1996) demonstrated that the formation of such an LBHB would weaken, rather than enhance, transition‐state solvation and would therefore exert an anticatalytic effect. In contrast, enzymes and polar solutions are more effective at stabilizing the transition state through localized charge interactions than through delocalized bonding interactions (Garcia‐Viloca et al., 2001). It is also important to emphasize that gas‐phase calculations that have been cited in support of the LBHB proposal (Warshel & Aqvist, 1991) are not relevant to enzyme active sites, where environmental effects dominate. All available EVB studies (see discussion in [Schutz & Warshel, 2004] as well as molecular‐orbital QM/MM studies that reach a quantitatively reliable level [Garcia‐Viloca et al., 2001; Molina et al., 2003; Mulholland et al., 2000]) contradict the LBHB hypothesis.

It is worth emphasizing at this point that, contrary to some implications in the literature (e.g., Weinhold, 1997), our analysis of LBHB (Warshel et al., 1995) is based on the empirical valence bond (EVB) framework, which is arguably the most reliable approach currently for examining environmental effects on covalent interactions and charge‐transfer contributions in hydrogen bonding. Within the EVB formulation, the diabatic states and covalent mixing elements are parameterized to reproduce ab initio ground‐state potential energy surfaces and the associated changes in charge distribution observed during gas‐phase reactions.

### 1.4 Electron transfer in proteins

An interesting issue that may confuse the argument about quantum mechanical effects is the issue of biological electron transfer (ET). In this case we would like to know how much ET is accelerated in biological systems relative to the same process in nonbiological media.

Here we start by considering the rate of ET. That is, as was shown in our initial work (Warshel, 1982) and subsequent works (Hwang & Warshel, 1985; Warshel & Hwang, 1986), one can obtain the rate of ET by using a semiclassical surface hopping approach. This involves running classical trajectories on the surface of the reactant and/or combination of the reactant and the product surfaces. While the surface hopping approach involves significant approximations, we find that at least for harmonic potential surfaces in the high temperature limit, the semiclassical expression converges to the exact quantum mechanical expression (Marcus & Sutin, 1985). In this limit, the saddle‐point approximation gives:

(1) $$k_{12}=\frac{\left(2\pi /\hbar \right){\left|\sigma_{12}\right|}^{2}}{\sqrt{4\pi \lambda k_{B}T}}\exp\left[-\frac{{\left({∆\epsilon }_{12}^{0}+\lambda \right)}^{2}}{4\pi \lambda k_{B}T}\right]$$

Here $∆\epsilon_{12}^{0}$ is the difference between the mean potential energies of the reactant and product states (⟨ϵ_2_⟩_2_ − ⟨ϵ_1_⟩_1_), and λ, the reorganization energy, is the energy required to change the system between the most probable configurations of the two states. From Equation (1) we find that the activation energy of the reaction ($∆\epsilon^{\neq }$) is:

(2) $$∆\epsilon^{\neq }=\frac{{\left({∆\epsilon }_{12}^{0}+\lambda \right)}^{2}}{4\lambda }$$

If we define the reaction coordinate *x* as the energy difference (*x* ≡ Δϵ_12_ = ϵ_2_ − ϵ_1_) and assume that the energy surfaces ϵ_1_ and ϵ_2_ are harmonic functions of this coordinate, the reorganization energy can be related to the displacement of the two minima.

(3) $$\lambda =\frac{1}{2}\left|{\left\langle ∆\epsilon_{12}\right\rangle }_{1}-{\left\langle ∆\epsilon_{12}\right\rangle }_{2}\right|$$

Replacing the potential energies Δϵ_12_, ${∆\epsilon }_{12}^{0}$, and $∆\epsilon^{\neq }$ by the corresponding free energies (ΔG_12_, ${∆G}_{12}^{0}$, and $∆G^{\neq }$) gives the well‐known Marcus equation (Marcus, 1956; Marcus & Sutin, 1985).

**FIGURE 3:**
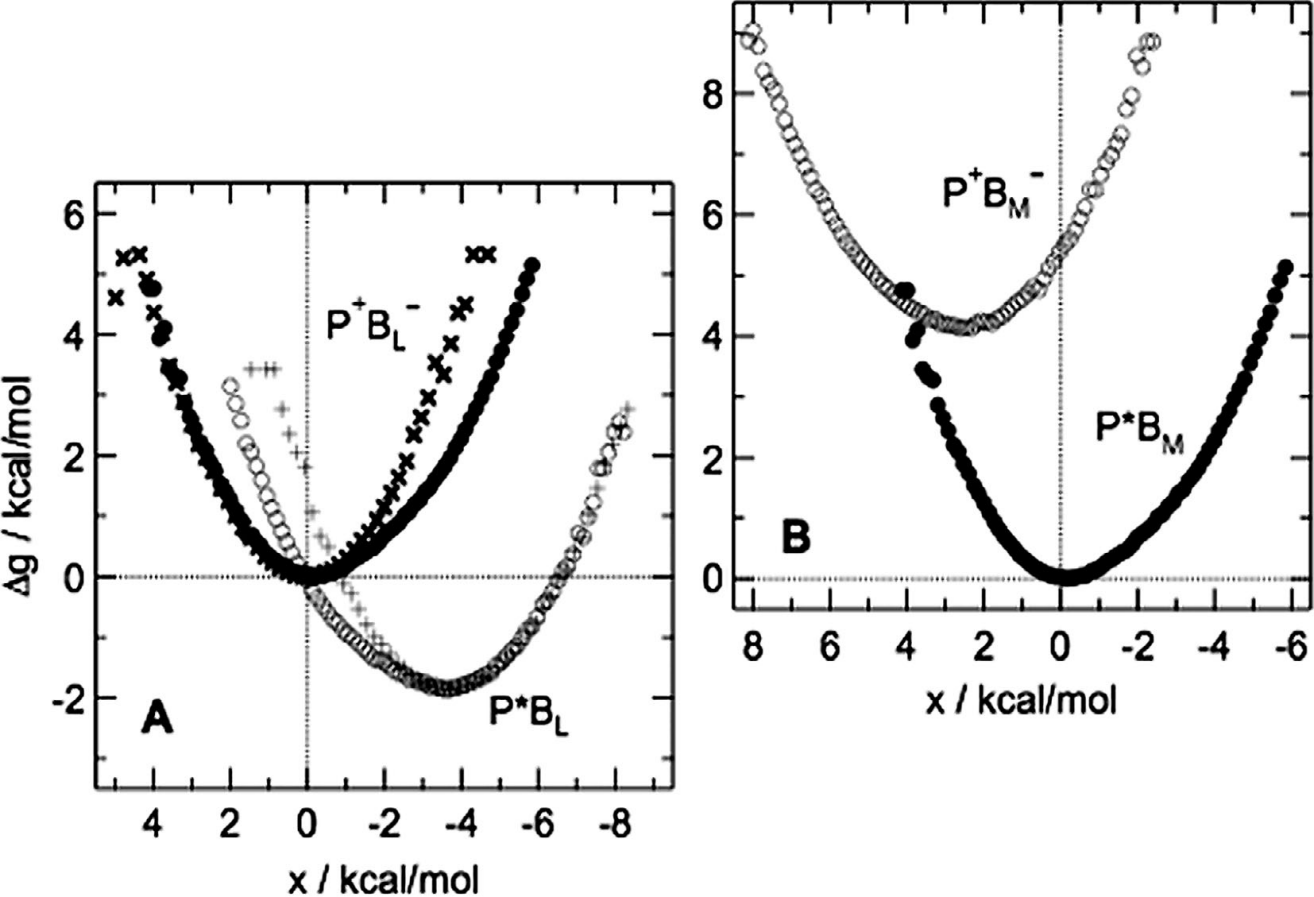
(a) Calculated free‐energy functions for the diabatic reactant (P*B_L_) and product (P^+^B_L_^−^) states in the reaction P*B_L_ → P^+^B_L_^−^ in *Rb. sphaeroides* reaction centers. The abscissa is the reaction coordinate, defined as the electronic energy difference between the two diabatic states. Free energy profiles obtained from separate ensemble sampling on each diabatic surface are shown by crosses (reactant) and plus symbols (product), whereas those obtained from the combined ensemble sampling are shown by filled circles (reactant) and open circles (product). The combined ensemble refers to configurations sampled from both diabatic states and reweighted to construct the free‐energy profiles. (b) Same as (a) but for the reaction P*B_M_ → P^+^B_M_^−^, where only the combined ensemble results are shown. Adapted from (Warshel & Parson, 2001).

Coming back to the issue of quantum effects in biology, we can start with the exponential factor. Here we can ask how proteins reduce the corresponding activation barriers. In this case, we have found that it is done by reducing the classical reorganization energy as we established for cytochrome c (Churg et al., 1983) and in the case of bacterial reaction centers (Warshel & Parson, 2001; Warshel & Schlosser, 1981). Thus the corresponding catalytic effect is not associated with a quantum effect. One can argue that in the so‐called inverted Marcus region, where Equation (1) is not valid and one has to consider transitions between vibronic channels (Warshel & Parson, 2001). In this case, it may be suggested that the inverted region blocks the very fast relaxation to the initial ground state. However, our study established that the vibronic channels allow a relatively fast relaxation to the ground state (Parson & Warshel, 2004b) and that only because the reorganization energy is tuned to be very similar to the free energy difference (see Figure 3) we have an optimal quantum yield (Parson & Warshel, 2004a).

Another factor that can be examined as a possible quantum effect is the possibility that preexponential electronic coupling, $\sigma_{12}$ in Equation 1, leads to quantum mechanical advantage. Here the opinions are different, where Gray and coworkers (Beratan et al., 1992) have promoted the idea of specific coupling paths, where Dutton and coworkers (Moser et al., 1992) have promoted a uniform distance dependance of coupling. Here again we have to consider the fact that the reference reaction in water also involves an electronic coupling.

Another direction where one may look for quantum mechanical effects is the limit where the surface hopping approach becomes invalid and one should move to a density matrix treatment. A density matrix approach based on actual microscopic simulations has been developed by Parson and Warshel (2004a, 2004b) and applied to bacterial reaction centers (Parson & Warshel, 2004a). This study, which is only relevant to very low temperatures, established that the ET in bacterial photosynthesis occurs before the excitation energy uses vibrational relaxations to decay to the minimum of the first excited state. This effect may be classified as a quantum mechanical non‐Marcus effect. However, this effect is not relevant at room temperature.

It is worth noting that in the Marcus inverted regime, quantum mechanical tunneling between vibronic states can, in principle, contribute to an acceleration of ET (Warshel, 1980). However, this situation is distinct from the cases of hydride or proton transfer discussed above, where the dominant catalytic effect arises from reduction of the activation barrier. In those systems, conditions that enhance tunneling are generally associated with higher barriers and therefore do not contribute to catalysis.

### 1.5 Antennas of reaction centers

An interesting case where long‐lived quantum coherence is likely to play a functional role in the antenna systems of photosynthetic reaction centers has been suggested by Fleming and coworkers (Ishizaki & Fleming, 2012) and others (Engel et al., 2007). This idea has been supported by experimental observations of short‐lived coherence. However, the biological relevance of such observations is far from clear.

First, antenna complexes operate in a warm, wet, and noisy biological environment. Thermal motions, protein vibrations, and solvent interactions lead to rapid decoherence, typically on femtosecond timescales. This makes sustained phase coherence across chromophores extremely difficult to maintain. Second, antenna pigments are strongly coupled to their surrounding protein scaffold and vibrational modes (phonons). This strong environmental coupling continuously disrupts phase relationships, causing energy transport to behave effectively as classical hopping rather than coherent wave‐like motion. Third, energetic disorder—both static and dynamic—dominates real antenna systems. Site‐energy fluctuations localize excitons and prevent the phase matching required for coherent transport across the antenna.

Importantly, near‐unity energy transfer efficiency can already be explained by classical models such as Förster resonance energy transfer and related open‐system approaches. Since these mechanisms are robust across temperatures and mutations, there is little evolutionary pressure for biology to rely on fragile quantum coherence.

While ultrafast spectroscopy does detect coherent oscillations, these signals are often better interpreted as vibronic coherence rather than functionally relevant electronic quantum coherence. In this sense, coherence may be an epiphenomenon of molecular structure rather than a biological design principle.

Finally, reaction center antenna systems appear optimized for robustness and reliability, not delicacy. In fact, environmental noise can enhance transport by preventing exciton trapping, a phenomenon sometimes described as environment‐assisted transport (Rebentrost et al., 2009). Crucially, this enhancement relies on the rapid suppression of coherence rather than its preservation. At any rate, the efficiency of photosynthesis is determined by the charge separation steps and not by the efficiency of the light absorption.

### 1.6 Vision

The primary event in vision involves an extremely efficient surface crossing with a quantum yield that approaches 1.0 (Warshel, 1976). Here it is tempting to invoke the involvement of quantum effects.

In analyzing this idea we introduced the first MD simulation of a biological process and the use of semiclassical surface hopping approach in biology (Warshel, 1976), This simulation predicted correctly the time scale and efficiency of the primary event. This study and subsequent works led to the realization that the effective surface crossing is associated with conical intersection (Olivucci et al., 1993; Warshel & Chu, 2001). Such effect can of course also occur in the reference solution process, but the relevant energy gap is far too large in solution. The protein, on the other hand, tunes the energy gap to a region where the surface crossing is optimized. However, as we have shown in (Schulten et al., 1978), the protein does not generate a perfect permanent conical intersection since its thermal fluctuations modulate the energy gap. Thus we are clearly not dealing with a quantum coherence. It seems to us that the main effect of the protein is to tune the energy gap by using electrostatic effects, not by quantum mechanical effects.

## 2 BIRD NAVIGATION AS AN EXAMPLE

### 2.1 Introduction

**FIGURE 4:**
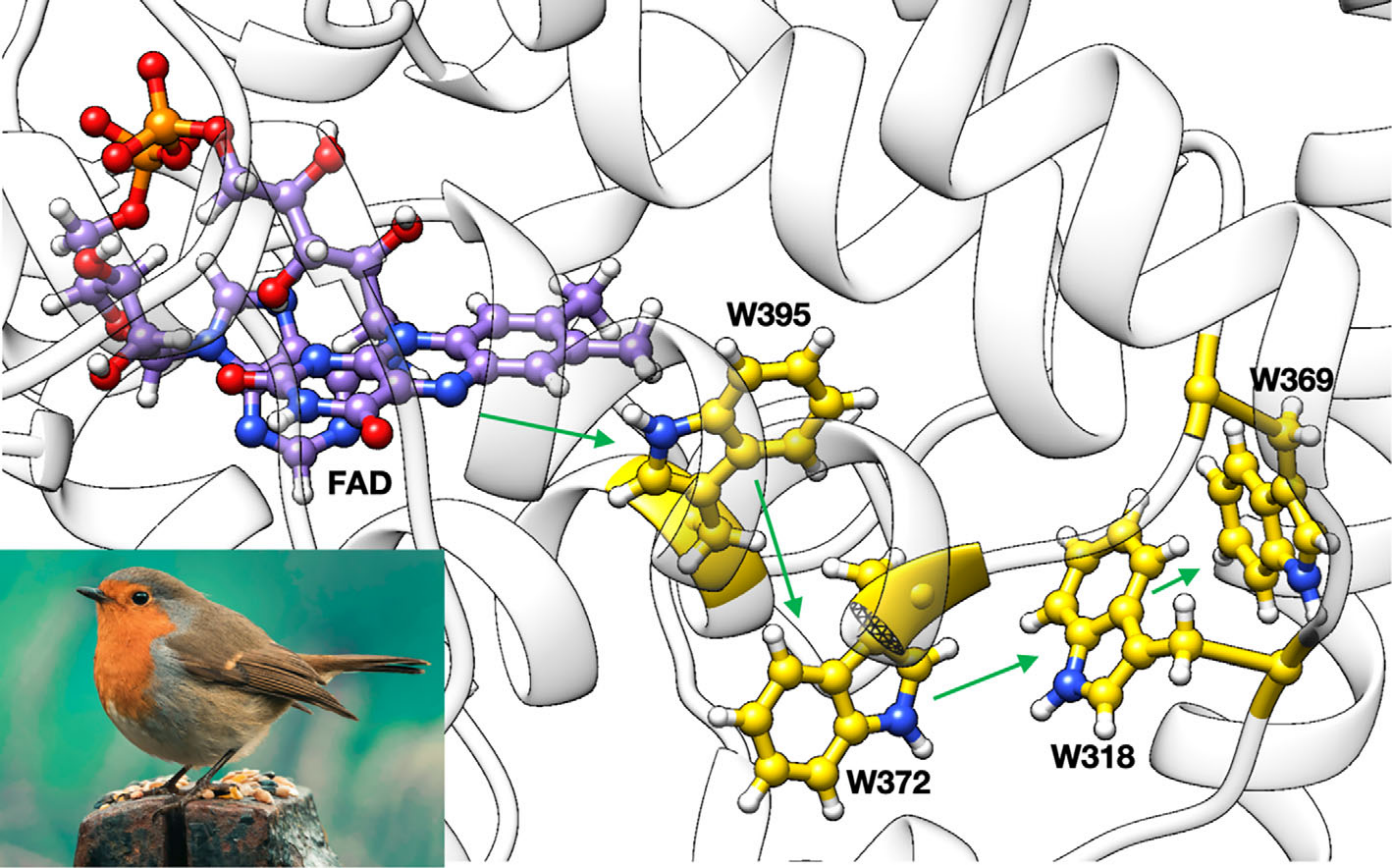
Structure of key residues and cofactors in ErCry4a. The flavin cofactor is in purple and the conserved tryptophan residues forming the ET chain are in gold, with green arrows indicating the photo‐induced electron transfer from FAD and the subsequent charge migration along the tryptophan chain. Inset: *Erithacus rubecula* (European robin).

Magnetoreception in migratory birds represents one of the most intriguing unsolved problems in sensory biology. Unlike vision (Wald, 1968; Warshel, 1976), olfaction (Buck & Axel, 1991), or mechanosensation (Ranade et al., 2015), whose molecular mechanisms are largely established, the biophysical origin of magnetic field detection remains under active investigation (Fleissner et al., 2007; Kirschvink et al., 2001; Schulten et al., 1978; Wiltschko & Wiltschko, 2005, Luo et al., 2024). Among the proposed mechanisms, the radical pair model (Ritz et al., 2000) has received substantial experimental and theoretical support, particularly through the work of Hore and co‐workers (Xu et al., 2021), who identified a cryptochrome of *Erithacus rubecula* (ErCry4a) as a potential magnetosensitive candidate. In this model, as shown in Figure 4, photoexcitation of the flavin adenine dinucleotide (FAD) cofactor initiates a sequence of ET steps along a conserved tryptophan chain, generating a spin‐correlated radical pair whose singlet–triplet interconversion can be influenced by weak magnetic fields (Hore & Mouritsen, 2016). The dim light present during the night might appear insufficient to modulate the behavior of nocturnal birds; however, moonlight spans a broad visible spectrum with decent intensity (Kieffer & Stone, 2005), making it capable of exciting the flavin chromophore. In addition, studies in small songbirds have shown that moonlight intensity correlates with their migratory behavior (Prinz et al., 2025).

Extensive theoretical efforts have been devoted to modeling both the ET energetics and the subsequent spin dynamics. QM/MM (Kattnig & Hore, 2017; Luo et al., 2025; Solov'yov et al., 2007) calculations have been employed to characterize charge localization and electronic couplings, while spin Hamiltonian and density‐matrix treatments (Hore & Mouritsen, 2016; Timmel et al., 1998) have been used to examine magnetic field effects under realistic hyperfine interactions. Despite these advances, the degree to which the elementary ET steps themselves rely on intrinsically quantum mechanical effects, beyond those associated with spin evolution, remains unclear.

Importantly, while the overall magnetoreception mechanism remains controversial, due to unresolved issues such as the identity of the signaling species, the detailed kinetics of the process, and the extent to which spin coherence can persist under physiological conditions (Hore & Mouritsen, 2016; Xu et al., 2021), the second and third ET steps along the tryptophan chain are comparatively well characterized experimentally (Timmer et al., 2023). Their kinetic timescales have been estimated from spectroscopic measurements, providing benchmarkable observables for theoretical analysis. These steps occur on sub‐nanosecond timescales following the initial photoinduced charge separation and are therefore critical in defining the lifetime and stability of the radical pair intermediate.

The availability of experimentally observed ET steps provides an opportunity to examine a more specific question: to what extent are these reactions governed by classical thermally activated processes versus delicate quantum mechanical effects associated with electronic coherence or nonadiabatic dynamics? In the present work, we analyze these two ET steps using EVB simulations combined with diabatic electronic coupling calculations, with the aim of assessing the validity of Marcus‐type behavior, the role of environmental linear response, and the robustness of the process under near‐ambient conditions.

### 2.2 Methods

#### 2.2.1 Initial PDB structure preparation

The protein structure was obtained by using sequence from (Xu et al., 2021) and predicted by AlphaFold Server (Jumper et al., 2021; Varadi et al., 2022). The predicted structure was superimposed with experimentally determined cryptochrome4 (PDB ID: 6PU0) (Zoltowski et al., 2019) for visual check and flavin cofactor binding pose determination.

#### 2.2.2 Ab‐initio calculation

The atomic charges for relevant molecular fragments were evaluated Electrostatic Potential (ESP)‐derived charges (Breneman & Wiberg, 1990), generated by Gaussian16 (Frisch et al., 2016) at the ωB97X‐D/6–311 + G(d,p) level (Chai & Head‐Gordon, 2008) with the Solvation Model based on Density (SMD) (Marenich et al., 2009). The restrained electrostatic potential (RESP)‐fitted charges (Bayly et al., 1993) for each fragment were then generated using AmberTools23 (Case et al., 2023). For these calculations, the tryptophan residue is truncated to beta carbon and cleaved bond saturated with a hydrogen atom. The flavin cofactor is truncated to lumiflavin and the side chain for the ab‐initio calculation.

Electronic couplings between charge‐localized diabatic states were computed using the constrained density functional theory configuration interaction (CDFT‐CI) (Kaduk et al., 2012) approach as implemented in Q‐Chem 6.2 (Epifanovsky et al., 2021). All calculations were performed at a fixed nuclear geometry using the range‐separated hybrid functional ωB97X‐D and the cc‐pVDZ basis set. Solvent effects were included via the integral equation formalism polarizable continuum model (IEF‐PCM) (Cancès et al., 1997) with the following dielectric parameters: static dielectric constant ε = 40 and optical dielectric constant ε_∞_ = 1.76.

In a first step, a constrained DFT (CDFT) (Wu & Van Voorhis, 2006) single‐point calculation was carried out to generate a solvent reaction field equilibrated to a specific diabatic charge‐localized state. A spin‐resolved charge constraint was imposed to localize one excess positive charge on the donor fragment, while the total system charge and spin multiplicity were fixed to represent an open‐shell radical cation. The converged PCM reaction field corresponding to this diabatic electronic state was saved and subsequently reused.

In a second step, CDFT‐CI calculations were performed using the frozen solvent reaction field obtained from the initial CDFT calculation, thereby eliminating contributions from solvent reorganization to the electronic coupling. Two diabatic states were constructed by imposing equivalent spin‐resolved charge constraints on the donor and acceptor fragments, respectively. Configuration interaction was then carried out in the subspace spanned by these diabatic states to obtain the diabatic Hamiltonian matrix, from which the electronic coupling was extracted as the off‐diagonal Hamiltonian element. This protocol ensures that the reported electronic couplings correspond to purely electronic interactions between charge‐localized states under a consistent solvent polarization.

#### 2.2.3 Monte Carlo sampling

The term ionization configuration (IC) is defined as the most probable protonation states of ionizable residues in a protein at a certain pH (Nandi et al., 2024). The IC of the studied protein system was obtained by combining intrinsic pKa calculation with PDLD/S‐LRA method and a Monte Carlo procedure (Sham et al., 1997; Singh & Warshel, 2010) for IC ensemble free energy sampling by:

(4) $${∆G}_{\mathrm{pH}}^{\left(m\right)}=-\sum_{i}1.38q_{i}^{\left(m\right)}\left({\mathrm{pKa}}_{\mathrm{int},i}^{p}-\mathrm{pH}\right)+166\sum_{i,j\neq i}\frac{q_{i}^{\left(m\right)}q_{j}^{\left(m\right)}}{r_{\mathrm{ij}}\varepsilon_{\mathrm{ij}}}$$

where ${∆G}_{\mathrm{pH}}^{\left(m\right)}$ denotes the free energy change of the protein transition from a hypothetical reference state in which all amino acids have a zero net charge, to the mth IC. $q_{i}^{\left(m\right)}$ denotes the net charge of the ith residue in the mth IC. ${\mathrm{pKa}}_{\mathrm{int},i}^{p}$ denotes the intrinsic pKa of the ith residue calculated by the PDLD/S‐LRA method (Sham et al., 1997). $r_{\mathrm{ij}}$ denotes the distance between the ith and the jth residue. $\varepsilon_{\mathrm{ij}}$ denotes a distance dependent dielectric constant to account for charge–charge interaction, defined as:

(5) $$\varepsilon_{\mathrm{ij}}=1+60\left(1-e^{-0.1r_{\mathrm{ij}}}\right)$$

${∆G}_{\mathrm{pH}}^{\left(m\right)}$ was sampled by Monte Carlo sampling and the ensemble averaged net charge of each residue can be estimated by:

(6) $${\left\langle q_{i}\right\rangle }_{\mathrm{pH}}=\frac{\sum_{m}q_{i}^{\left(m\right)}\exp\left(-\beta ∆G_{\mathrm{pH}}^{\left(m\right)}\right)}{\sum_{m}\exp\left(-\beta ∆G_{\mathrm{pH}}^{\left(m\right)}\right)}$$

#### 2.2.4 PDLD/S‐LRA method

**FIGURE 5:**
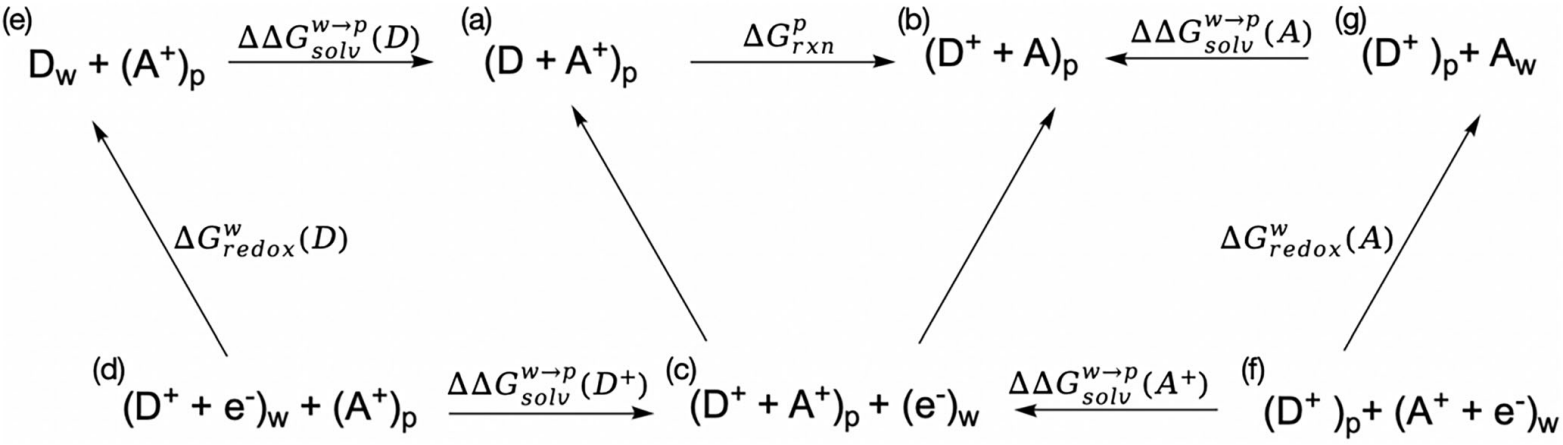
Thermodynamic cycle for evaluation of electron transfer free energy in the protein environment. D and A refer to electron donor and acceptor, respectively, with subscript to indicate the environment (w: bulk water, p: protein). (e^−^)_w_ denotes a hypothetical electron donor reservoir in the bulk water but does not directly interact with the system of our interest. The free energies terms calculated are presented on the arrows connecting different states and consistent with the arrow directions. The target reaction is from state (a) to state (b) with free energy change of ${∆G}_{\mathrm{rxn}}^{p}$. ${∆∆G}_{\text{solv}}^{w\to p}$ terms refer to the corresponding solvation free energy differences by moving the fragment from water to the protein environment. ${∆G}_{\text{redox}}^{w}$ terms refer to the corresponding redox free energy in bulk water. By combining the aqueous redox terms with the corresponding solvation free energy differences, the protein‐phase reaction free energy is obtained. In the present system, where ET occurs between two tryptophan residues, the intrinsic aqueous redox contributions cancel, and ${∆G}_{\mathrm{rxn}}^{p}$ is determined primarily by environmental polarization effects.

The ET free energy between the donor and the acceptor was calculated by the following thermodynamic cycle presented in Figure 5. The solvation free energy differences were calculated by the PDLD method within the LRA regime (Sham et al., 2000), and the free energy of aqueous redox reactions should in principle be obtained by experimental measurement or ab initio calculations. However, in the present study, the contributions of aqueous reactions cancel since the ET happens between two tryptophan residues.

**FIGURE 6:**
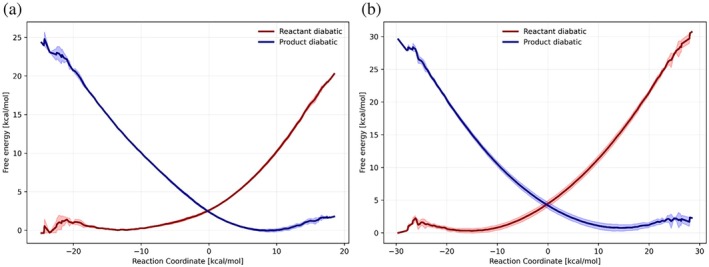
EVB calculations of the electron transfer reactions in the tryptophan chains. (a) and (b) describe the second and the third electron transfer, respectively. Each figure describes the free energy functionals of the corresponding diabatic states (reactant state in red, and product state in blue). Solid lines represent the average free energy values and the corresponding shaded areas refer to the standard deviation.

In order to calculate the ET free energy inside the protein environment, four PDLD calculations are needed to obtain the solvation free energy of each residue in the corresponding redox state. The in‐water properties can either be calculated by high level ab initio or from experiments. In the case of the present study, the in‐water contributions cancel since the ET is between two tryptophans.

The initial phase configuration and momentum of the systems were from the corresponding MD relaxations, with all ionizable residues assigned half of their ensemble averaged charge calculated by the MC procedure (Sham et al., 1997; Singh & Warshel, 2010). The electrostatic component of the free energy difference was calculated by averaging 20 configurations sampled in a 20 ps simulation with an effective protein dielectric constant ε_p_ = 20 to account for the reorganization effect that is implicit in the semi‐microscopic treatment (Schutz & Warshel, 2001; Sham et al., 1998; Singh & Warshel, 2010).

#### 2.2.5 EVB method

The EVB method was used to get the corresponding reaction free energy profiles using the MOLARIS‐XG package (Warshel et al., 2012). In these simulations, the reactive region (region 1) includes the donor and acceptor tryptophans, and the remaining protein‐solvent system was assigned to region 2. The system was solvated in a water sphere of radius 20 Å surrounded by Langevin dipoles up to 22 Å and simulated under Surface Constrained All Atom Solvent (SCAAS) boundary conditions (Warshel & King, 1985), with long‐range electrostatics treated using the local reaction field approach (Lee & Warshel, 1992).

The system was first equilibrated in their reactant state starting from 5.0 K gradually to 274.15 K in a 40 ps simulation with time step size of 1fs, and then another 40 ps simulation on the target temperature has been implemented for the initial phase space sampling of replica for the EVB simulations. The EVB simulations were started from the five independent replicas from relaxation. Each EVB simulation consisted of 31 frames with 10 ps simulation per umbrella sampling window. The EVB Hamiltonian parameters were then obtained by matching the reaction free energies calculated in the thermodynamic cycle by the PDLD/S‐LRA‐2000 method (Sham et al., 2000).

### 2.3 Results

#### 2.3.1 Electronic coupling sensitivity

The electronic coupling terms in Marcus formula were obtained by CDFT‐CI calculations as shown in Table 1. Since the protein environment is highly heterogeneous, the CDFT‐CI calculations were done in IEF‐PCM implicit solvation model with various dielectric constants to check the sensitivity of electronic coupling to the polarizability of the environment. It is observed that the electronic coupling is more sensitive to which diabatic electronic state the solvent is interacting with than to the polarizability of the environment. For such reason, we used the electronic couplings calculated with the dielectric constant of 40 for the following Marcus theory calculations.

**TABLE 1:** Electronic coupling of the second electron transfer from CDFT‐CI^a^.

Solv_field^b^	Dielectric constants^c^						
	4	6	8	20	40	60	80
	|Hab|						
rct	0.000593	0.0005996	0.0006026	0.0006074	0.0006087	0.0006091	0.0006093
pdt	0.0004066	0.0003835	0.0003704	0.0003434	0.0003334	0.0003299	0.0003282

^a^ Electronic coupling terms are in the unit of Hartree.

^b^ Solvent field used in the calculation, rct and pdt refer to the IEF‐PCM responses to the reactant and product diabatic electronic states, respectively.

^c^ Dielectric constants used for the hypothetical bulk solvent.

#### 2.3.2 EVB free energy profile

Our analysis with careful EVB calculations of the second and the third ET reactions (i.e., ET from Trp372 to Trp395, and from Trp318 to Trp372). The results are summarized in Table 2 and shown in Figure 5. The protonation configurations at pH 7.0 and the detailed electron‐transfer free energies from the PDLD calculations are summarized in Tables S1–S3. The corresponding ET rate constants were calculated by Marcus theory (Equation 1). The corresponding reorganization energies and activation free energies were directly from EVB profiles, and the electronic coupling is obtained using CDFT‐CI calculations averaged over the reaction field equilibrated in the reactant and product diabatic states.

**TABLE 2:** Calculated Marcus parabolas of the electron transfer reactions and the corresponding observed results^a^.

Reaction	ΔG_0_ ^b^	λ_fw_	λ_bw_	ΔG^≠^	τ_calc_ ^c^	τ_exp_ ^d^
ET2	−0.06	8.47	13.18	2.51	22.0	30
ET3	0.42	14.70	13.73	4.04	158.2	141

^a^ All energies are in the unit of kcal mol^−1^ and characteristic times in ps.

^b^ Reaction free energies were obtained by the corresponding Thermodynamic cycle shown in Figure 6.

^c^ Characteristic time constants are estimated by the inverse of the corresponding rate constants obtained by the EVB profiles and Marcus theory.

^d^ Observed values from^75^.

### 2.4 Discussion

The present study examined the second and third ET steps along the tryptophan chain using a combination of EVB free energy simulations and diabatic electronic coupling calculations. Both reactions are experimentally benchmarkable and occur on sub‐nanosecond timescales, making them among the fastest processes in the system aside from the initial photoinduced charge separation. The calculated free energy profiles exhibit near‐parabolic behavior and yield activation barriers and characteristic times in good agreement with experiment. This agreement indicates that an ensemble‐based free energy description on Born–Oppenheimer potential surfaces is sufficient to capture the essential thermodynamics and kinetics of these steps.

The EVB results further suggest that the solvent and protein environment follow linear response behavior. The forward and backward reorganization energies are comparable in magnitude, and the free energy surfaces are well described by the Marcus formalism. Within this framework, the transition region corresponds to an intermediate polarization state located approximately midway between the reactant and product minima. This observation is consistent with the validity of the linear response approximation under the near‐ambient temperature conditions considered here (274.15 K).

Electronic coupling calculations performed under solvent reaction fields equilibrated separately to the reactant and product diabatic states reveal significant differences between these polarization limits. However, the arithmetic mean of the two limiting couplings yields rate constants that are in good agreement with experiment. This behavior is naturally explained within the Marcus picture: if the solvent response is approximately linear and the coupling varies smoothly with the reaction field, the physically relevant coupling at the crossing region is expected to lie between the two limiting values. The success of the averaged coupling therefore reflects the smooth dependence of the electronic interaction on environmental polarization, rather than requiring explicit trajectory‐based nonadiabatic simulations in which the solvent polarization must be assigned to a partially mixed electronic state along the trajectory. In polarizable environments, determining a physically consistent reaction field corresponding to a coherent superposition of diabatic states is formally ambiguous and often requires additional decoherence corrections. The present approach avoids this ambiguity by evaluating the coupling under well‐defined polarization limits associated with fully localized diabatic states. Importantly, the coupling shows only weak sensitivity to the magnitude of the dielectric constant, indicating that permanent electrostatic polarization, rather than fine‐tuned dielectric response, governs its variation.

The calculated forward and backward ET rates are of comparable magnitude, implying that reversible charge transfer may occur on timescales similar to those of radical pair spin evolution. Such reversible dynamics would lead to stochastic modulation of the spin Hamiltonian through fluctuations in exchange, dipolar, and hyperfine interactions associated with the changing charge distribution. From the perspective of open quantum systems, time‐dependent modulation of the Hamiltonian by nuclear motion is a well‐established source of dephasing. Thus, even without invoking additional mechanisms, thermally driven charge fluctuations may contribute to limiting electronic spin coherence under near‐ambient conditions. A quantitative assessment of this effect would require explicit spin‐dynamics simulations coupled to stochastic ET kinetics and is beyond the scope of the present study.

Taken together, the results indicate that the elementary ET steps in the tryptophan chain operate within a classical Marcus regime characterized by thermally activated barrier crossing, near‐Gaussian environmental response, and smoothly varying electronic interactions. No evidence is found for fragile or finely tuned quantum mechanical control of the charge transfer process itself. If magnetoreception arises from radical pair spin dynamics within this framework, its functional behavior must therefore emerge from collective spin evolution embedded in a thermally fluctuating protein environment, rather than from delicate quantum fine‐tuning at the level of individual molecular interactions.

## 3 CONCLUDING REMARKS

The cases considered here indicate that quantum mechanical effects do not play a major role in biology.

We start by pointing out that enzyme catalysis does not involve quantum mechanical effects, not in the form of tunneling enhanced catalysis nor in the form of more exotic effects.

We also consider the possible involvement of quantum mechanical effects in photosynthesis and vision. Here we conclude that also in such light induced systems, proteins operate their functions at room temperature by classical effects.

## AUTHOR CONTRIBUTIONS

**Aoxuan Zhang:** Conceptualization; investigation; writing – original draft; methodology; validation; visualization; writing – review and editing; formal analysis; data curation. **Arieh Warshel:** Conceptualization; investigation; funding acquisition; writing – original draft; methodology; validation; visualization; writing – review and editing; software; formal analysis; data curation; supervision; resources.

## FUNDING INFORMATION

This work was supported by National Science Foundation Grant MCB‐1707167 and National Institute of Health Grant R35 GM122472.

## CONFLICT OF INTEREST STATEMENT

The authors declare no competing interests.

## Supporting information


**Data S1.** Supporting information.

**Table S1.** Ionization configuration from Monte Carlo proton transfer method.
**Table S2.** PDLD results for the second electron transfer reaction in ErCry4a^a^.
**Table S3.** PDLD results for the third electron transfer reaction in ErCry4a^a^.

## Data Availability

The data that support the findings of this study are available from the corresponding author upon reasonable request.
