# Supplementary material for: How important are quantum mechanical effects in controlling biological functions: Enzymes, electron transfer and bird navigation
Source: Protein Sci. 2026 Jun 19;35(7):e70683. doi: 10.1002/pro.70683 (PMC13282187; doi:10.1002/pro.70683)
Supplement: Supplementary file 2 — Table S1. Ionization configuration from Monte Carlo proton transfer method. Table S2. PDLD results for the second electron transfer reaction in ErCry4aa. Table S3. PDLD results for the third electron transfer reaction in ErCry4aa. [file PRO-35-e70683-s001.docx]

Supplementary information to:

**How Important are Quantum Mechanical Effects in Controlling Biological Functions: Enzymes, Electron Transfer and Bird Navigation**

Aoxuan Zhang and Arieh Warshel*

**Table S1**: Ionization configuration from Monte Carlo proton transfer method.

residue# = the position of that residue

name = amino acid type

config = ensemble averged net charge

pKa_int = intrinsic pKa

| residue# | name | config | pKa_int |
| --- | --- | --- | --- |
| 17 | ASP | -0.997 | 7.55 |
| 38 | ASP | -1 | 2.4 |
| 62 | ASP | -1 | 2.23 |
| 87 | ASP | -1 | 6.52 |
| 100 | ASP | -0.001 | 4.01 |
| 135 | ASP | -1 | 2.69 |
| 141 | ASP | -0.999 | 5.03 |
| 163 | ASP | -1 | 5.15 |
| 175 | ASP | -1 | 3.57 |
| 183 | ASP | 0 | 3.89 |
| 185 | ASP | -0.999 | 4.44 |
| 197 | ASP | -1 | 4.99 |
| 225 | ASP | -1 | 3.74 |
| 321 | ASP | -1 | 2.64 |
| 339 | ASP | -1 | 0.3 |
| 367 | ASP | -1 | 5.91 |
| 385 | ASP | -0.95 | 11 |
| 387 | ASP | -1 | 4.41 |
| 421 | ASP | -1 | 0.74 |
| 463 | ASP | -1 | 5.15 |
| 476 | ASP | -1 | 5.7 |
| 498 | ASP | -1 | 5.08 |
| 499 | ASP | -1 | 6.04 |
| 501 | ASP | -1 | 4.62 |
| 502 | ASP | -0.866 | 5.18 |
| 511 | ASP | -1 | 5.72 |
| 12 | GLU | -0.999 | 7.29 |
| 29 | GLU | -0.997 | 7.69 |
| 61 | GLU | -0.999 | 6.4 |
| 80 | GLU | -0.002 | 3.79 |
| 82 | GLU | -0.946 | 4.97 |
| 102 | GLU | -1 | 4.44 |
| 104 | GLU | -0.997 | 4.42 |
| 109 | GLU | -0.55 | 4.04 |
| 111 | GLU | -0.993 | 4.82 |
| 120 | GLU | -1 | 5.13 |
| 124 | GLU | -0.995 | 6.44 |
| 165 | GLU | -1 | 6.35 |
| 174 | GLU | -1 | 4.91 |
| 188 | GLU | -1 | 6.21 |
| 203 | GLU | -1 | 4.55 |
| 212 | GLU | -0.997 | 3.12 |
| 214 | GLU | -0.998 | 7.29 |
| 220 | GLU | -0.999 | 7.73 |
| 292 | GLU | -1 | 5.87 |
| 323 | GLU | -1 | 5.41 |
| 348 | GLU | -0.994 | 7.45 |
| 373 | GLU | -1 | 3.41 |
| 374 | GLU | -1 | 5.63 |
| 380 | GLU | -0.993 | 8.38 |
| 381 | GLU | -1 | 5.06 |
| 444 | GLU | -1 | 5.26 |
| 450 | GLU | -1 | 4.72 |
| 451 | GLU | -0.962 | 4.48 |
| 452 | GLU | -1 | 3.33 |
| 473 | GLU | -0.964 | 6.12 |
| 487 | GLU | -1 | 7.63 |
| 488 | GLU | -0.999 | 5.71 |
| 505 | GLU | -1 | 6.15 |
| 514 | GLU | -1 | 4.45 |
| 515 | GLU | -1 | 4.93 |
| 527 | GLU | -1 | 5.36 |
| 11 | LYS | 0.995 | 3.66 |
| 65 | LYS | 1 | 9.43 |
| 91 | LYS | 1 | 8.95 |
| 108 | LYS | 1 | 10.78 |
| 152 | LYS | 1 | 10.23 |
| 199 | LYS | 0.999 | 10.45 |
| 234 | LYS | 0.735 | 8.34 |
| 275 | LYS | 0.988 | 10.27 |
| 320 | LYS | 1 | 9.43 |
| 327 | LYS | 1 | 9.82 |
| 329 | LYS | 0.712 | 6.98 |
| 377 | LYS | 1 | 8.85 |
| 418 | LYS | 1 | 7.88 |
| 429 | LYS | 1 | 10.24 |
| 435 | LYS | 0.607 | 10.47 |
| 440 | LYS | 1 | 7.81 |
| 454 | LYS | 1 | 9.05 |
| 472 | LYS | 1 | 10.12 |
| 507 | LYS | 0.175 | 10.76 |
| 509 | LYS | 0.999 | 6.27 |
| 519 | LYS | 1 | 9.98 |
| 521 | LYS | 0.002 | 8.92 |
| 27 | SER | 0 | 21.22 |
| 28 | SER | 0 | 24.03 |
| 44 | SER | 0 | 15.8 |
| 45 | SER | 0 | 24.66 |
| 59 | SER | 0 | 22.41 |
| 72 | SER | 0 | 19.46 |
| 93 | SER | 0 | 20.16 |
| 127 | SER | 0 | 15.87 |
| 130 | SER | 0 | 21.09 |
| 132 | SER | 0 | 17.76 |
| 146 | SER | 0 | 17.75 |
| 159 | SER | 0 | 20.27 |
| 180 | SER | 0 | 18.44 |
| 201 | SER | 0 | 15.37 |
| 206 | SER | 0 | 22.53 |
| 231 | SER | 0 | 18.87 |
| 241 | SER | 0 | 18.26 |
| 245 | SER | 0 | 18.14 |
| 250 | SER | 0 | 22.95 |
| 254 | SER | 0 | 22.46 |
| 259 | SER | 0 | 18.08 |
| 268 | SER | 0 | 20.3 |
| 278 | SER | 0 | 20.38 |
| 283 | SER | 0 | 19.98 |
| 299 | SER | 0 | 20.47 |
| 371 | SER | 0 | 16.59 |
| 389 | SER | 0 | 22.5 |
| 399 | SER | 0 | 19.69 |
| 401 | SER | 0 | 16.18 |
| 439 | SER | -0.082 | 17.48 |
| 449 | SER | 0 | 15.02 |
| 475 | SER | 0 | 24.64 |
| 518 | SER | 0 | 17.16 |
| 3 | HIS | 0.997 | 4.35 |
| 7 | HIS | 0 | -2.91 |
| 16 | HIS | 0.994 | 1.96 |
| 64 | HIS | 0.869 | 3.21 |
| 88 | HIS | 0.999 | 3.87 |
| 131 | HIS | 0.959 | 1 |
| 156 | HIS | 0 | 5.96 |
| 222 | HIS | 0.998 | 8.59 |
| 276 | HIS | 0 | 6.86 |
| 277 | HIS | 0.406 | 7.07 |
| 326 | HIS | 0.033 | 6.02 |
| 353 | HIS | 0 | 1.95 |
| 357 | HIS | 0.119 | 4.38 |
| 405 | HIS | 0 | 2.6 |
| 471 | HIS | 0.999 | 2.72 |
| 477 | HIS | 0.999 | 5.62 |
| 490 | HIS | 0 | 5.11 |
| 512 | HIS | 0 | 4.97 |
| 4 | ARG | 1 | 13.53 |
| 10 | ARG | 0.994 | 5.86 |
| 14 | ARG | 1 | 6.88 |
| 39 | ARG | 1 | 10.4 |
| 52 | ARG | 1 | 11.41 |
| 86 | ARG | 1 | 9.42 |
| 138 | ARG | 1 | 11.46 |
| 153 | ARG | 1 | 11.62 |
| 169 | ARG | 1 | 7.04 |
| 178 | ARG | 1 | 13.51 |
| 191 | ARG | 1 | 12.02 |
| 209 | ARG | 1 | 13.72 |
| 218 | ARG | 0.997 | 11.58 |
| 236 | ARG | 0.356 | 11.77 |
| 261 | ARG | 1 | 10.45 |
| 266 | ARG | 1 | 11.38 |
| 291 | ARG | 1 | 10.23 |
| 324 | ARG | 1 | 10.57 |
| 346 | ARG | 1 | 12.13 |
| 356 | ARG | 0.95 | 5.72 |
| 365 | ARG | 1 | 9.79 |
| 409 | ARG | 1 | 9.74 |
| 415 | ARG | 1 | 9.58 |
| 419 | ARG | 0.995 | 11.45 |
| 428 | ARG | 0.996 | 10.64 |
| 462 | ARG | 1 | 11.48 |
| 483 | ARG | 1 | 12.06 |
| 486 | ARG | 1 | 10.36 |
| 491 | ARG | 1 | 12.06 |
| 497 | ARG | 1 | 11.97 |
| 510 | ARG | 1 | 12.18 |
| 524 | ARG | 1 | 11.71 |
| 47 | HIE | 0.993 | 6.13 |
| 54 | HIE | 0.551 | 4.25 |
| 352 | HIE | 0 | -5.28 |
| 32 | TYR | 0 | 14.81 |
| 35 | TYR | 0 | 13.84 |
| 81 | TYR | 0 | 9.22 |
| 107 | TYR | 0 | 13.35 |
| 134 | TYR | 0 | 16.36 |
| 151 | TYR | 0 | 17.84 |
| 190 | TYR | -0.027 | 13.54 |
| 252 | TYR | 0 | 18.65 |
| 265 | TYR | 0 | 17.42 |
| 271 | TYR | -0.333 | 14.11 |
| 295 | TYR | 0 | 14.58 |
| 319 | TYR | -0.161 | 13.76 |
| 388 | TYR | 0 | 13.3 |
| 407 | TYR | -0.342 | 12.44 |
| 426 | TYR | 0 | 20.33 |
| 430 | TYR | -0.006 | 18.27 |
| 441 | TYR | 0 | 11.64 |
| 443 | TYR | 0 | 13.53 |
| 464 | TYR | 0 | 12.86 |
| 68 | CYS | -0.25 | 12.15 |
| 73 | CYS | -0.034 | 11.13 |
| 116 | CYS | 0 | 9.95 |
| 179 | CYS | 0 | 12.76 |
| 189 | CYS | 0 | 9.75 |
| 257 | CYS | 0 | 11.35 |
| 313 | CYS | 0 | 15.22 |
| 317 | CYS | 0 | 8.96 |
| 361 | CYS | -0.999 | 10.58 |
| 412 | CYS | -1 | 5.69 |
| 458 | CYS | 0 | 9.7 |

**Table S2:** PDLD results for the second electron transfer reaction in ErCry4a^a^.

| cmp | c1 | ddG_elec(20) | c2 | ddG_nonelec | dG_bind | dG_et |
| --- | --- | --- | --- | --- | --- | --- |
| D0372 | 0.5 | 0.18 | 0.25 | 0.38 | 0.56 | -0.06 |
| D0395 | 0.5 | 0.6 | 0.25 | -0.8 | -0.2 |  |
| S0372 | 0.5 | -0.11 | 0.25 | 0.04 | -0.07 |  |
| S0395 | 0.5 | -0.15 | 0.25 | -0.74 | -0.89 |  |

^a^All free energy quantities have the unit of kcal/mol

cmp = compound

c1 = coefficient for electrostatic component of the binding free energy

ddG_elec(20) = electrostatic component of the binding free energy with dielectric constant e_p = 20

c2 = coefficient for non-electrostatic component of the binding free energy

dG_bind = binding free energy

dG_et = forward electron transfer reaction free energy

D0372 = one-electron oxidized tryptophan 372

D0395 = one-electron oxidized tryptophan 395

S0372 = normal tryptophan 372

S0395 = normal tryptophan 395

**Table S3:** PDLD results for the third electron transfer reaction in ErCry4a^a^.

| cmp | c1 | ddG_elec(20) | c2 | ddG_nonelec | ddG_nonelec_rescale | dG_bind | dG_et |
| --- | --- | --- | --- | --- | --- | --- | --- |
| D0318 | 0.5 | 0.95 | 0.25 | 0.38 | 0.38 | 1.33 | 0.68 |
| D0372 | 0.5 | 0.6 | 0.25 | -0.8 | -0.8 | -0.2 |  |
| S0318 | 0.5 | 0.09 | 0.25 | 0.04 | 0.04 | 0.13 |  |
| S0372 | 0.5 | 0.02 | 0.25 | -0.74 | -0.74 | -0.72 |  |

^a^All free energy quantities have the unit of kcal/mol

cmp = compound

c1 = coefficient for electrostatic component of the binding free energy

ddG_elec(20) = electrostatic component of the binding free energy with dielectric constant e_p = 20

c2 = coefficient for non-electrostatic component of the binding free energy

dG_bind = binding free energy

dG_et = forward electron transfer reaction free energy

D0318 = one-electron oxidized tryptophan 318

D0372 = one-electron oxidized tryptophan 372

S0318 = normal tryptophan 318

S0372 = normal tryptophan 372
